# Risk factors for injury in a national cohort of 87,134 Thai adults

**DOI:** 10.1016/j.puhe.2011.09.027

**Published:** 2012-01

**Authors:** V. Yiengprugsawan, K. Stephan, R. McClure, M. Kelly, S. Seubsman, C. Bain, A.C. Sleigh, Thai Cohort Study Team

**Affiliations:** a National Centre for Epidemiology and Population Health, The Australian National University, Canberra, Australia; b Monash University Accident Research Centre, Monash University, Melbourne, Australia; c School of Human Ecology, Sukhothai Thammathirat Open University, Nonthaburi, Thailand; d School of Population Health, The University of Queensland, Brisbane, Australia

**Keywords:** Risk factors, Transport injury, Home injury, Work injury, Sport injury, Thailand

## Abstract

**Background:**

Information is needed regarding risk factors associated with injury. In middle- and lower-income countries, injury studies have focused on road traffic injuries and less attention has been given to other types of injuries.

**Methods:**

This study is part of overarching health–risk transition research in Thailand with a large national cohort study that began in 2005 (*n* = 87,134). Associations between potential determinants and overall injury were measured, as well as injury by location (transport, home, work and sport), using data gathered from the baseline questionnaire.

**Results:**

In total, 21.5% of the cohort reported at least one incidence of injury over the last 12 months. Risk factors associated with injury were being male [odds ratio (OR) 1.20], having lower income (OR 1.70), having problems with vision (OR 1.46), having epilepsy (OR 3.02), having depression/anxiety (OR 1.62), poor self-assessed health (OR 1.68), being obese (OR 1.24) and death of father due to injury (OR 1.59). Analysis of injury by location provided more specific information on risk factors. For example, females were more likely to report injuries at home, while males, urban residents and regular alcohol drinkers were more likely to report transport injuries.

**Conclusions:**

The risk of injury in Thailand varies considerably by location, sociodemographic group and several categories of co-existing morbidities. Such epidemiological information identifying risk factors for injury is useful for designing targeted injury prevention programmes in Thailand and other middle-income countries.

## Background

Injury in developing and transitional economies is an important public health problem. Indeed, injury accounts for 9% of global mortality, with the majority of these injury deaths occurring in lower- and middle-income countries, resulting in both social and economic loss.^1–3^ For example, in a report from the USA, almost one-fifth of the population received medical treatment for injury over 1 year.^4^ Injuries can occur anywhere, and can include transport injuries, occupation-related injuries in the agricultural or industrial sectors, injuries at home or injuries while playing sport.^5–7^ Studies in many developing countries have focused on road traffic injuries and less attention has been given to other types of injuries.^8–10^

The health response to injury involves delineating the nature of the problem, identifying risk factors, developing countermeasures, and evaluating the programmes that will help to identify key population-based strategies for injury prevention.^11,12^ To date, there is limited information on demographic, physical, behavioural and social risk factors of injuries in the developing world.^13,14^ Indeed, a population-based understanding of injury could help to address a problem of growing international importance.

As part of an ongoing analysis of the health-risk transition in Thailand, a large Thai cohort study began in 2005. The 12-month incidence of injuries recalled at baseline by cohort members has been reported elsewhere, with injury frequency broken down by sociodemographic attributes, injury mechanisms and intentionality.^15^ Briefly, nearly 22% of cohort members reported at least one injury over the previous 12 months. Most injuries were unintentional.

In a separate report, transport-related injuries reported by cohort members were analysed, noting the frequency, distribution, behavioural determinants, types of vehicles and roles of the injured persons.^16^ Overall, 8.4% of respondents reported a transport-related injury, with higher risks for younger males using motorcycles. Drink driving was more common among males. The usage rates of motorcycle helmets and seatbelts were higher than previously reported for Thailand.

The present study analysed the baseline injury results further by testing for relationships with various risk factors. This study had three objectives: (1) to compare the incidence of injuries among the cohort by injury location, sociodemographic attributes, physical and mental health, and health behaviours; (2) to measure associations between risk factors and injury; and (3) to compare risk factors for injury by location of occurrence.

## Methods

### Data source

The Thai Health-Risk Transition research project includes adult distance-learning students from Sukhothai Thammathirat Open University (STOU), resident throughout Thailand. The cohort was recruited in 2005 from 200,000 STOU students. A 20-page questionnaire was posted to each student and 87,134 (44%) responded. Details on cohort enrolment and overall methodology have been reported elsewhere.^17,18^ Briefly, the STOU cohort is representative of the geodemographic, ethnic and socio-economic status (income and assets) of the adult Thai population. Using the results of the 2000 Population and Housing Survey, the median age was 29.2 years for the Thai population and 29.0 years for cohort members, and 51% of the Thai population were females compared with 54% of cohort members.^17^ As the cohort members are well educated, they are able to respond to complex health questionnaires, including questions regarding the occurrence and circumstances of injuries.

This study analysed the baseline questionnaire from 2005, which includes information about injury in the previous 12 months and a wide array of potentially associated demographic, socio-economic, behavioural and health factors. Data scanning, and verification and editing of questionnaire data were undertaken using Scandevet, a program developed by a research team from Khon Kaen University. Final data editing was undertaken using SQL and Statistical Package for the Social Sciences (SPSS Inc., Chicago, IL, USA).

### Measures

The core injury question was ‘In the last 12 months, how many injuries have you had that were serious enough to interfere with daily activities and/or required medical treatment?’ The injury threshold was set at this level to capture ‘minor’ injury which is known to constitute a large proportion of the population injury burden. These criteria for injury reporting threshold have been widely accepted.^19^ Additional questions provided the locales of injuries coded as home, workplace, sport and transport. Other variables chosen for this analysis included potential determinants of injuries and associated potential confounders of injury associations [age, sex, marital status, socio-economic status, area of residence, body mass index (BMI), vision, hearing, epilepsy, self-assessed health, anxiety, death of father or mother due to injury, and certain behaviours including drinking alcohol, smoking and exercise].

### Definitions

Age groups (years) were divided into five bands: 15–19, 20–29, 30–39, 40–49 and ≥50 years. Marital status was classified as not married or married. The geographic and socio-economic indicators used were urban or rural residence, and income was divided into four groups (≤7000, 7001–10,000, 10,001–20,000 and >20,000 Baht/month (1 USD = 40 Baht in 2005). Physical health assessments included self-reported problems with vision (two binary variables – yes/no for ‘need glasses or contact lenses’and yes/no for ‘problems uncorrectable by glasses or contact lenses’) or hearing (responses collapsed to ‘good’ or ‘some trouble/deaf’). In addition, cohort members were asked if they had ever been diagnosed with ‘epilepsy’ or ‘depression/anxiety’ by a doctor. Self-assessed overall health was based on the first question of the Short Form 8 Medical Outcomes Instrument for which six response categories were collapsed to ‘poor/very poor’ and ‘not poor’ (i.e. fair/good/very good/excellent). BMI was calculated from reported height and weight, and classified by Asian standards as underweight (<18.5 kg/m^2^), normal (18.5–22.9 kg/m^2^), overweight at risk (23–24.9 kg/m^2^) and obese (≥25 kg/m^2^). Health behaviours included smoking and drinking, and both were analysed on the basis of current status as ‘yes’ or ‘no’. The reported weekly frequency of at least moderate exercise (breathe harder than normal) for >20 min was categorised into three groups: ‘none’, ‘1–2 times per week’ and ‘≥3 times per week’.

### Statistical analysis and model selection

Individuals with missing data for certain analyses were excluded so the totals varied slightly according to the information available. Analysis for the association between injury outcome and determinants was undertaken with Stata software using multivariate logistic regression, reporting odd ratios (OR) and *P*-values based on two-tailed tests. For initial logistic regression modelling, overall injury was used as an outcome (‘0’ if no injury in the past 12 months, ‘1’ if reporting at least one injury that was serious enough to interfere with daily activities and/or required medical attention). Model 1 included sociodemographic variables, Model 2 included sociodemographic and measured physical variables (vision, hearing, epilepsy, anxiety, self-assessed overall health, BMI, parental death due to injury), and Model 3 included Model 2 as well as certain behavioural variables (drinking alcohol, smoking and exercise). Thus, Model 3 was fully adjusted to prevent confounding.

For location-specific injury outcomes, all the explanatory variables included in Model 3 were tested in a series of analyses for each of the four location-categorized injuries (transport, home, work and sport).

## Results

### Overall injuries and their sociodemographic, physical and behavioural determinants

#### Injuries by location and sex

Injuries were common among the STOU students, and 21.5% of the cohort reported at least one injury over the past 12 months (Table 1). Repeated injury was much less common, with 3.6% of the cohort reporting two injuries, 1.2% reporting three injuries and 1.1% reporting more than three injuries over the past 12 months (data not shown). The incidence rates of various types of injuries were as follows: falls (4.4%), other blunt force (2.8%), stab or cut (2.4%), fire or heat (0.5%), assault (0.4%) and poisoning (0.3%).

The highest proportion of locale-specific injury was transport-related (8.3%), especially for males (9.9%). Home injuries were more prominent among females (6.2% compared with 4.3% among males). The opposite was true for sport injuries (1.0% for females compared with 5.2% among males).

#### Sociodemographic characteristics

More cohort members were female (54.7%), and 44.9% of males and 60.9% of females were aged <30 years. There were more non-married cohort members, especially among females (62.9%). Using income as a socio-economic measure, 42.0% of the cohort reported earning <7000 Baht/month; 47.4% of the females in the cohort were in this low-income category. In total, 48.3% of cohort members were living in urban areas.

#### Physical health of the cohort members

Almost 8% of the cohort had problems with vision that could not be corrected by glasses or contact lenses, and 24% of males and 31.4% of females needed glasses or contact lenses. Approximately 8% of both males and females reported hearing problems. Less than 0.3% of the cohort reported that they had been diagnosed with epilepsy, and 3.5% reported that they had been diagnosed with anxiety; the proportions were similar for males and females. In terms of BMI, 21.8% of females and 6.2% of males were underweight, 9.7% of females and 21.7% of males were overweight, and 9.9% of females and 22.7% of males were obese. Family history of death by injury was also considered: 2.6% of males and 3.1% of females reported that their father had died due to injury, and 4.8% and 5.7% reported that their mother had died due to injury. It should be noted that 73.2% of the cohort reported that their father was still alive, and 88.1% reported that their mother was still alive (data not shown).

#### Health-related behaviours of the cohort members

Alcohol drinking was included as a potential determinant of injuries. Less than 1% of females reported regular drinking, whereas close to 10% of males were regular drinkers. Moderate exercise more than 3 times per week was reported by 37.6% of males and 24.7% of females.

### Potential determinants of overall injury over the past 12 months

Three models associating overall injury with all potential determinants were tested (Table 2). Across the three models, being male and having a lower income were significantly associated with overall injury. In the final model, variables that were significantly positively associated with overall injury were problems with vision (OR 1.46), epilepsy (OR 3.02), depression/anxiety (OR 1.62), poor self-assessed overall health (OR 1.68), being obese (OR 1.24, and death of father due to injury (OR 1.59).

### Potential determinants of injuries over the past 12 months by location

Table 3 presents potential explanatory variables for location-specific injuries including transport, home, work and sport. Sociodemographically, males were more likely than females to have transport (OR 1.24) and sport (OR 4.32) injuries, but were less likely to have injuries at home (OR 0.71). Lower income was strongly associated with transport (OR 2.18) and work-related (OR 2.02) injuries. Cohort members living in urban areas were more likely to report transport injuries (OR 1.41) and injuries at home (OR 1.32) than those living in rural areas.

Non-correctable problems with vision were associated with all injury location categories with ORs ranging from 1.38 to 1.86. Epilepsy was strongly associated with injuries at home (OR 3.90). Increasing anxiety was associated with transport injuries (OR 1.64), and poor or very poor self-assessed health was positively associated with all injury categories. Obesity was associated with transport injuries (OR 1.34). All injury categories were associated with death of father due to injury (ORs 1.45 to 1.69), and this was significant (*P* < 0.05) for transport injuries. Drinking alcohol was significantly and positively associated with transport injuries (OR 1.68 for regular drinkers). Exercising more than three times per week was associated with increased risk of injury, especially in the case of sport injuries (OR 2.20).

## Discussion

Injury is an important component of the health-risk transition as a country undergoes socio-economic development. The Thai Cohort Study reported injury and its potential determinants retrospectively at baseline in 2005. Injuries were frequent, affecting more than one-fifth (21.5%) of the cohort members each year. This rate was similar to that noted in a comprehensive US review, which found that approximately 20% of 15–44 year olds had injuries that required medical treatment each year.^4^ This US study used a similar threshold for recording injury.

Analysis of injury by location (transport, home, work and sport) provided more specific information on risk factors. For example, in the study cohort, transport injuries were the most common and affected 8.3% of respondents per year. These transport injuries had a dose-response relationship with increasing poverty, and were more common among males and urban residents. Other reports have noted that traffic accidents are important contributors to national disease burdens in both developed and developing countries.^20,21^ In agreement with the present study, several other publications have noted that drinking alcohol is one of the main behavioural determinants of transport-related injuries, especially among younger males.^22–24^ Elsewhere, the authors have reported that drink driving among males was common (40–60%, varying by age group), and regular use of motorcycle helmets never exceeded 70%.^16^ These findings on risk factors for transport injuries are important for Thailand. A recent publication based on Thai National Injury Surveillance reports noted that mortality in transport accidents was dramatically higher when seatbelts or motorcycle helmets were not used.^25^

Low income was one of the main risk factors for work injuries in the study cohort, and this phenomenon has been noted elsewhere in Asia. For example, a study of almost 25,000 people in Vietnam emphasized the role of poverty in association with injuries. Poverty was found to increase the risk of injury at home for children and elderly people, and to increase the risk of work-related injuries for adults (15–59 years).^26^ Interestingly, another study of work-related injury among 15,000 Vietnamese respondents reported that non-agricultural work was considerably more hazardous than agricultural work, with the implication that injuries will increase as Vietnam develops.^27^ It is assumed that this process is further advanced in Thailand, as the proportion of the Thai workforce engaged in agriculture is declining.^28^

Several studies in the USA, Europe and Australia have reported details on the determinants of injuries at home. Being female and having poor eyesight have been found to be significant predictors of injury-causing falls, while home repairs, gardening and car care were more often associated with injuries among males.^29–32^ The present study found that epilepsy was a major determinant of injury (especially at home), and this supports the results of a study of eight countries in Europe. This latter study followed epileptic cases and matched non-epileptic controls for nearly 17,500 person-months, and found that those with epilepsy reported more domestic accidents, more hospitalisation and more medical complications compared with the controls.^33^

Sport injuries were the least common category in the study cohort, but did affect 5.2% of males per year. Such injuries are commonly reported in other settings.^6,34,35^ Among Canadian adolescents and young adults, sport is one of the main causes of injuries requiring medical attention; area of residence, weekly hours of exercise and increased BMI were all predictors of sport injuries in adolescents.^35^ In the present study, exercising more than three times per week was strongly related with sport injuries, but BMI and area of residence were not risk factors.

Other interesting risk factors were also apparent in this analysis. For example, when history of parental death due to injury was analysed separately for males and females, the association between the respondent’s injury and paternal death and the non-association between the respondent’s injury and maternal death were found to be independent of the sex of the respondent (data not shown). Also of interest was the finding that frequent alcohol intake had an inverse (protective) association with injury at home; this phenomenon was more pronounced when analysis was restricted to males. These observations could reflect increased absence from home for regular drinkers, but no data are available to test such an explanation. Other data (not shown) are supportive as males who reported regular drinking were about five times more likely to report frequent social interactions with friends and colleagues. Finally, poor self-assessed health was positively associated with injury in all locations, but the authors could not determine which came first as an association is plausible in either direction (i.e. injury could cause poor self-assessed health or vice versa).

High injury rates can place a substantial social and economic burden on lower- and middle-income countries. Information generated by surveillance data and epidemiological studies of injury is needed to design effective preventive programmes. Some possible interventions include modification of home environments or sporting facilities for the reduction of injuries.^36^ For certain population subgroups, strategies should be directed at both the environment and at individual factors, especially for injuries which occur at home.^37,38^

A feature of this study is the self-reporting method used to detect injuries. The recall period adopted (12 months) is similar to that used in many other population studies of injury. Due to the better education of the respondents and the non-use of proxies for reporting, the data are probably less affected by underestimation problems that are normally encountered in the injury statistics for developing countries. A possible limitation of this study is due to the nature of 1-year retrospective cohort analysis, which may cause some problems with reverse causation between determinants and injury outcomes. As the cohort progresses prospectively, it will be possible to examine further exposure outcomes in a longitudinal context. This paper has addressed some knowledge gaps on factors associated with injuries, and offers insights which could be useful for injury prevention programmes in Thailand and other middle-income countries.

## Ethical approval

Sukhothai Thammathirat Open University Research and Development Institute (Protocol 0522/10) and the Australian National University Human Research Ethics Committee (Protocol 2004344). Informed written consent was obtained from all participants.

## Funding

This study was supported by the International Collaborative Research Grants Scheme with joint grants from the Wellcome Trust UK (GR071587MA) and the Australian NHMRC (268055), and a global health grant from the NHMRC (585426).

## Competing interests

None declared.

## Figures and Tables

**Table 1:** Incidence of injury and distribution of potential risk factors: cohort members, 2005.

Injuries and potential risk factors	% of cohort members injured			
*n* = 87,134	*n*	Males	Females	All
*Injury by location in the past 12 months*	18,731	24.8	18.8	21.5
Transport injury	4995	9.9	7.0	8.3
Home injury	7277	4.3	6.2	5.3
Work injury	3384	4.9	3.1	3.6
Sports injury	2529	5.2	1.0	2.9

Characteristics of cohort members	% of cohort		
Males	Females	All
*Sociodemographic characteristics*			
Female			54.7
Age 15–19 years	2.2	3.5	2.9
Age 20–29 years	42.7	57.4	50.7
Age 30–39 years	34.8	28.5	31.4
Age 40–49 years	16.4	9.4	12.6
≥50 years	3.9	1.3	2.5
Marital status: married	51.1	37.1	43.4
Income ≤7000 Baht/month	35.4	47.4	42.0
Income 7001–10,000 Baht/month	22.9	23.6	23.3
Income 10,001–20,000 Baht/month	28.4	20.8	24.2
Income >20,000 Baht/month	13.4	8.2	10.5
Residence: urban	46.7	53.3	48.3
*Physical and mental health status*			
Sight: need glasses or contact lenses	24.0	31.4	28.0
Sight: not correctable by glasses or lenses	7.9	7.8	7.9
Hearing: some trouble	8.2	8.8	8.6
Epilepsy (ever diagnosed)	0.3	0.2	0.2
Depression/anxiety (ever diagnosed)	3.3	3.6	3.4
Self-assessed health: poor/very poor	3.8	5.2	4.6
Underweight (BMI <18.5 kg/m^2^)	6.2	21.8	14.7
Overweight at risk (BMI 23–24.9 kg/m^2^)	21.7	9.7	15.1
Obese (BMI ≥25 kg/m^2^)	22.7	9.9	15.7
Father died due to injury	2.6	3.1	2.8
Mother died due to injury	4.8	5.7	5.2
*Health-related behaviours*			
Current regular alcohol drinker	9.9	0.7	4.9
Current regular smoker	31.4	1.1	12.3
Exercise: none	36.1	56.4	47.3
Exercise: 1–2 times/week	26.3	24.7	25.4
Exercise: ≥3 times/week	37.6	18.9	27.3

BMI, body mass index.

1 USD = 40 Baht in 2005.

**Table 2:** Odds ratios and statistical significance for potential risks associated with overall injury during the past 12 months among Thai cohort members, 2005.

Potential risk factors	Model 1	Model 2	Model 3
*Sociodemographic characteristics*			
Female	1	1	1
Male	1.50∗∗∗	1.40∗∗∗	1.20∗∗
Age 15–19 years	1	1	1
Age 20–29 years	1.06	1.05	1.01
Age 30–39 years	1.05	0.85	0.84
Age 40–49 years	1.14∗	0.81	0.79
Age ≥50 years	1.20∗	0.81	0.72
Marital status: married	1	1	1
Marital status: not married	1.03	1.13	1.16
Income ≤7000 Baht/month	1.65∗∗∗	1.93∗∗∗	1.70∗∗∗
Income 7001–10,000 Baht/month	1.46∗∗∗	1.47∗∗∗	1.32∗
Income 10,001–20,000 Baht/month	1.23∗∗∗	1.28∗∗	1.17
Income >20,000 Baht/month	1	1	1
Residence: rural	1	1	1
Residence: urban	1.07∗∗∗	1.11	1.11
*Physical and mental health*			
Sight: need glasses or contact lenses		1.13	1.05
Sight: not correctable by glasses or lenses		1.64∗∗∗	1.46∗∗∗
Hearing: some trouble		1.15	1.15
Epilepsy (ever diagnosed)		2.82∗	3.02∗
Depression or anxiety (ever diagnosed)		1.50∗∗	1.62∗∗
Self-assessed health: fair and above		1	1
Self-assessed health: poor or very poor		1.60∗∗∗	1.68∗∗∗
Normal weight (BMI 18.5–22.9 kg/m^2^)		1	1
Underweight (BMI <18.5 kg/m^2^)		0.88	0.84
Overweight at risk (BMI 23–24.9 kg/m^2^)		1.06	1.08
Obese (BMI ≥25 kg/m^2^)		1.22∗∗	1.24∗
Father died due to injury		1.63∗∗	1.59∗
Mother died due to injury		0.94	0.93
*Health-related behaviours*			
Not a current regular alcohol drinker			1
Current regular alcohol drinker			1.14
Not a current regular smoker			1
Current regular smoker			1.33
Exercise: none			1
Exercise: 1–2 times/week			1.07
Exercise: ≥3 times/week			1.16

BMI, body mass index.

Note: ∗*P* < 0.05, ∗∗*P* < 0.01 and ∗∗∗*P* < 0.001. Model 1 is adjusted for all sociodemographic characteristics, Model 2 adds adjustments for physical and mental health, and Model 3 is adjusted for all attributes listed.

1 USD = 40 Baht in 2005.

**Table 3:** Odds ratios and statistical significance for potential risks associated with injuries during the past 12 months by location of occurrence.

Potential determinants	Transport	Home	Work	Sport
*Sociodemographic characteristics*				
Male	1.24∗	0.71∗	1.46∗	4.32∗∗∗
Age 20–29 years	0.95	0.83	0.65	0.88
Age 30–39 years	0.76	0.76	0.56	0.79
Age 40–49 years	0.79	0.73	0.51	0.47
Age ≥50 years	0.98	0.75	0.26∗	0.50
Marital status: married	1.29∗	0.84	1.38	0.93
Income ≤7000 Baht/month	2.18∗∗∗	1.36	2.02∗∗	1.25
Income 7001–10,000 Baht/month	1.63∗∗	0.96	1.67∗	1.00
Income 10,001–20,000 Baht/month	1.43∗	1.02	0.99	0.98
Residence: urban	1.41∗∗∗	1.32∗	0.82	1.05
*Physical and mental health*				
Sight: need glasses or contact lenses	0.89	1.15	1.06	1.05
Sight: not correctable by glasses	1.86∗∗∗	1.38∗	1.57∗	1.83∗∗
Hearing: some trouble	1.05	1.08	1.32	1.08
Epilepsy (ever diagnosed)	1.50	3.90∗	1.34	3.68
Depression or anxiety (ever diagnosed)	1.64∗	1.48	1.59	1.44
Self-assessed health: poor or very poor	1.64∗∗	1.40	1.98∗	1.80∗
Underweight (BMI <18.5 kg/m^2^)	1.13	0.73	0.89	0.41
Overweight at risk (BMI <23–24.9 kg/m^2^)	0.98	1.14	0.83	1.10
Obese (BMI ≥25 kg/m^2^)	1.34∗	1.11	1.25	0.93
Father died due to injury	1.69∗	1.56	1.54	1.45
Mother died due to injury	1.22	0.81	0.96	0.38
*Health-related behaviours*				
Current regular alcohol drinker	1.68∗	0.46	1.43	0.75
Current regular smoker	1.28	1.27	1.37	0.84
Exercise: 1–2 times/week	1.24	1.06	0.94	1.32
Exercise: ≥3 times/week	1.30∗	0.98	1.02	2.20∗∗∗

Note: ∗*P* < 0.05, ∗∗*P* < 0.01 and ∗∗∗*P* < 0.001. All odds ratios and *P*-values are derived from adjusted multivariate logistic regression based on Model 3 (see Table 2).

1 USD = 40 Baht in 2005.
